# Anoikis-related signature predicts prognosis and characterizes immune landscape of ovarian cancer

**DOI:** 10.1186/s12935-023-03170-8

**Published:** 2024-02-03

**Authors:** Jiani Yang, Yue Zhang, Shanshan Cheng, Yanna Xu, Meixuan Wu, Sijia Gu, Shilin Xu, Yongsong Wu, Chao Wang, Yu Wang

**Affiliations:** 1 Department of Gynecology, Shanghai First Maternity and Infant Hospital, School of Medicine, Tongji University, Shanghai, China; 2 Shanghai Key Laboratory of Maternal Fetal Medicine, Shanghai Institute of Maternal-Fetal Medicine and Gynecologic Oncology, Shanghai First Maternity and Infant Hospital, School of Medicine, Tongji University, Shanghai, 200092 China; 3 Department of Obstetrics and Gynecology, Renji Hospital, School of Medicine, Shanghai Jiaotong University, Shanghai, China

**Keywords:** Anoikis, Prognostic signature, Ovarian cancer, Immune landscape, DAPK1

## Abstract

Ovarian cancer (OV) is the most lethal gynecological malignancy worldwide, with high recurrence rates. Anoikis, a newly-acknowledged form of programmed cell death, plays an essential role in cancer progression, though studies focused on prognostic patterns of anoikis in OV are still lacking. We filtered 32 potential anoikis-related genes (ARGs) among the 6406 differentially expressed genes (DEGs) between the 180 normal controls and 376 TCGA-OV samples. Through the LASSO-Cox analysis, a 2-gene prognostic signature, namely AKT2, and DAPK1, was finally distinguished. We then demonstrated the promising prognostic value of the signature through the K-M survival analysis and time-dependent ROC curves (p-value < 0.05). Moreover, based on the signature and clinical features, we constructed and validated a nomogram model for 1-year, 3-year, and 5-year overall survival, with reliable prognostic values in both TCGA-OV training cohort (p-value < 0.001) and ICGC-OV validation cohort (p-value = 0.030). We evaluated the tumor immune landscape through the CIBERSORT algorithm, which indicated the upregulation of resting Myeloid Dendritic Cells (DCs), memory B cells, and naïve B cells and high expression of key immune checkpoint molecules (CD274 and PDCD1LG2) in the high-risk group. Interestingly, the high-risk group exhibited better sensitivity toward immunotherapy and less sensitivity toward chemotherapies, including Cisplatin and Bleomycin. Especially, based on the IHC of tissue microarrays among 125 OV patients at our institution, we reported that aberrant upregulation of DAPK1 was related to poor prognosis. Conclusively, the anoikis-related signature was a promising tool to evaluate prognosis and predict therapy responses, thus assisting decision-making in the realm of OV precision medicine.

**Supplementary Information:**

The online version contains supplementary material available at 10.1186/s12935-023-03170-8.

## Introduction

Ovarian cancer (OV), one of the most lethal gynecological malignancies, largely threatens the safety and health of women worldwide [1]. In the United States, there were 19,710 new cases and 13,270 deaths related to OV, which was estimated for 2023 [2]. Due to the lack of specific symptoms, approximately 70% of OV patients were diagnosed at advanced stages, with invasion of peripheral organs and metastasis, which lead to a poor 5-year overall survival (OS) rate of only 30% [3]. After the standard treatment of surgery combined with platinum-based chemotherapy, almost 70% of OV patients suffer cancer recurrence [4]. Accordingly, further studies are urgently needed to identify reliable prognostic biomarkers for personalized treatment.

Anoikis, a programmed cell death induced by cell detachment from the extracellular matrix (ECM), is one of the hallmarks of tumor metastatic skills, which attracts great attention from the scientific community [5]. Growing evidence demonstrated that anoikis could prevent adherent-independent cell growth, thus avoiding tumor implantation into ectopic sites where the ECM proteins are different [6]. Accordingly, failure to execute anoikis could enable adherent cells to survive under suspension conditions and proliferate at distant organs [7]. Anoikis is related to several mechanisms in cancer cells, including change in integrins' repertoire, upregulation of key enzymes involved in growth factor receptor signaling, and activation of a plethora of inside-out pro-survival signals [8]. The tumor microenvironment could also regulate the anoikis process of tumor cells by enhancing oxidative stress, modulating matrix stiffness, producing pro-survival soluble factors, and leading to metabolic deregulations, etc. [8, 9]. Several studies reported that anoikis played an essential role in tumor invasion and metastasis among malignancies including prostate cancer [10], lung cancer [11], pancreatic cancer [12], and breast cancer etc. [13]. However, few researchers have focused on the prognostic value of anoikis signature for OV patients and the underlying mechanism is still unclear.

Therefore, in our study, we identified and validated an anoikis-related signature (including AKT2 and DAPK1) to evaluate prognosis, predict immunotherapy/chemotherapy response, and guide clinical treatment for OV patients. We further validated the signature among OV patients through the IHC of tissue microarrays, PCR, and Western Blot analysis. We aimed to construct an anoikis-related signature as a candidate tool to evaluate prognosis, facilitate clinical management, and constitute promising targets for anti-metastatic pharmacological therapy for OV patients.

## Methods

### Publicly available databases and preprocessing

**Fig. 1:**
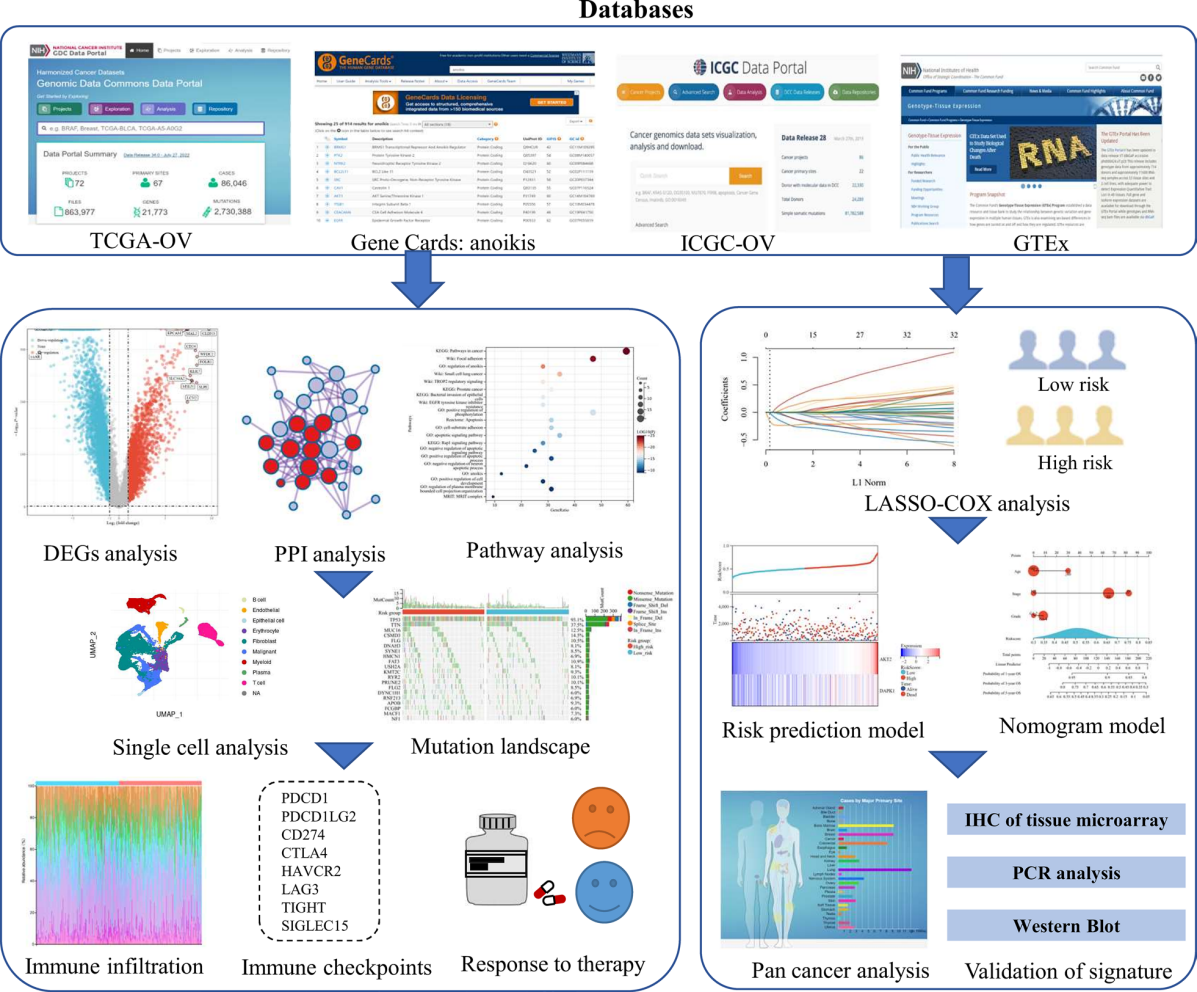
The flowchart of the research

The flowchart of the research was listed in Fig. 1. We downloaded both RNA-sequencing (RNA-seq) information and corresponding clinical characteristics from the Cancer Genome Atlas dataset (TCGA; https://portal.gdc.com) as a training cohort and from the International Cancer Genome Consortium dataset (ICGC; https://dcc.icgc.org) as a validation cohort. Transcriptome data of normal ovarian tissues were also retrieved from the GTEx dataset (https://gtexportal.org) as controls. To preprocess the data, we normalized raw count data through the “limma” package (R software, Version 4.2.0) and converted probes into corresponding gene symbols refer to the platform annotation file. Moreover, we identified the differentially-expressed genes (DEGs) between OV tissues and normal controls through the cut-off criteria of |Log2 (fold change)|> 1 the adjusted p-value < 0.05.

### Identification of differently expressed anoikis-related genes and somatic mutation analysis

Based on the GeneCards website (https://www.genecards.org), we obtained anoikis-related genes (ARGs, Relevance Score ≥ 2) by searching the term “anoikis”. Then, we selected ARGs, which were differentially expressed between normal controls and OV tissues according to the Venn diagram. To confirm underlying functions related to the filtered differentially expressed anoikis-related genes (DE-ARGs), we conducted the Gene Ontology (GO) and Kyoto Encyclopedia of Genes and Genomes (KEGG) enrichment analyses via the "ClusterProfiler" package (R software, Version 4.2.0). Stepwise, in order to provide hints for protein interactions network of DE-ARGs, we conducted the protein–protein interaction (PPI) analysis through the Metascape (https://metascape.org/), a database that could provide screens for a PPI network [14].

To evaluate the relationships between anoikis subtypes and clinicopathological features (including age, pathological grade, and clinical stage), we conducted the Sankey diagram via the “ggalluvial” package (R software, Version 4.2.0). We obtained the data of somatic mutations from the Genomic Data Commons and visualize the somatic landscape of OV cohort via the Oncoplot using the “maftools” package (R software, Version 4.2.0).

### Construction and validation of the anoikis-related signature

We conducted the Least Absolute Shrinkage and Selection Operator (LASSO)—COX algorithm with tenfold cross-validation, in order to identify prognostic ARGs for the signature construction, via the "glmnet" package (R software, Version 4.2.0). To estimate the prognosis value of the filtered DE-ARGs, we applied the Log-rank test and Kaplan–Meier (K-M) curves analysis. We also performed the Receiver Operating Characteristic curve (ROC) analysis for 1-year, 3-year, and 5-year OS rates, via the "timeROC" package (R software, Version 4.2.0). Moreover, according to the anoikis-related signature and corresponding clinical features, we conducted both univariate and multivariate Cox Regression algorithms to select independent risk factors of OV prognosis. Based on the filtered features, we constructed a prognostic nomogram for 1-year, 3-year, and 5-year OS rates via the “rms” package (R software, Version 4.2.0).

### Single-cell analysis

According to the Gene Expression Omnibus (GEO) database (https://www.ncbi.nlm.nih.gov/), we downloaded the raw data of the single-cell transcriptome profiling from Qian and colleagues [16], which contained 5 OV patients and 2 controls. We utilized the “Seurat” package (R software, Version 4.2.0) to filter out cells with poor quality. We then conducted standard data preprocessing, including calculating the percentage of gene numbers, evaluating cell counts, and analyzing mitochondria sequencing count. Next, we disregarded genes with less than 3 detected cells and excluded those cells with less than 200 detected genes. The reading depth was 10 × genomics, while the proportion of mitochondria was restricted to less than 5%. With logFC = 0.5 and p-value = 0.05 as the cutoff criteria, we utilized the t-SNE algorithm to perform cluster classification analysis and screened marker genes between clusters via the “Seurat” and “SingleR” packages (R software, Version 4.2.0). Afterward, we visualized the pseudo-time analysis via the “monocle” package (R software, Version 4.2.0). The single-cell analysis was conducted through the TIGER website (http://tiger.canceromics.org/).

### Evaluation of immune landscape and drug sensitivity

To obtain a brief view of the tumor immune landscape, we verified the abundance percentage of the 22 typical immune cells infiltrated in the tumor, based on the CIBERSORT algorithm [12]. Moreover, to predict patient response towards immunotherapy, we evaluated gene expression distribution for 8 typical immune checkpoints, including CTLA4, CD274, LAG3, HAVCR2, SIGLEC15, PDCD1, PDCD1LG2, and TIGIT, between the two risk groups classified by the anoikis-related signature through the Pearson's test. Based on the TIDE database (http://tide.dfci.harvard.edu), we evaluated the potential response of OV patients toward Immune Checkpoint Blockade (ICB) therapies. Additionally, based on the PRJEB23709 database [3], a tumor cohort treated with anti-CTLA-4 and anti-PD-1 therapies, we evaluated the prediction value of the anoikis-related signature among tumor patients treated with immunotherapies.

Moreover, we downloaded drug sensitivity data and genomic markers of drug response from the Genomics of Drug Sensitivity in Cancer datasets (GDSC, https://www.cancerrxgene.org), which is one of the largest pharmacogenomics datasets. Stepwise, we predicted the half-maximal inhibitory concentration values (IC50) of individuals through the Ridge Regression via the "pRRophetic" package (R software, Version 4.2.0), in order to evaluate OV patient response towards typical chemotherapies.

### Immunohistochemistry evaluation

Firstly, tissue samples were de-waxed, followed by the hydration and wash procedure. After microwave antigen retrieval, the sections were then treated with 3% H2O2 to block endogenous peroxidase activity. For the immunohistochemistry (IHC) analysis, we sequentially incubated the slides overnight with Anti- DAPK1 antibody (ABclonal, A5741, 1:50) and Mouse Anti-Rabbit IgG secondary antibody (ABclonal, AS061, 1:100). Stepwise, we counter-stained the signals by diaminobenzidine and hematoxylin. Two experienced pathologists scored the signal percentage and intensity of the slides, without any information about patients. They graded the staining intensity with a four-tier scale (0 = absent; 1 = weak; 2 = moderate; and 3 = strong). They graded the proportion of labeled tumor cells with a four-tier scale: 0 to 5% is 0 points, 6% to 25% is 1 point, 26% to 50% is 2 points, 51% to 75% is 3 points, and > 75% is 4 points. Immunoreactive score (IRS) was then determined based on the proportion of labeled tumor cells and the intensity of nuclear staining: IRS-Score = staining intensity * proportion of labeled tumor cells. A higher IRS-score (maximum score, 12) was defined as a higher expression [15].

### RT-PCR analysis

According to the manufacturer’s instructions, we extracted the total RNA samples using the Trizol Reagent (Sangon, B610409) and reverse transcribed RNA into cDNA using the RevertAid 1st Strand cDNA Synthesis Kit (ThermoFisher Scientific, K1622). Subsequently, the real-time quantitative reverse transcription-polymerase chain reaction (RT-PCR) analysis was conducted through the SYBR Green Mix (ThermoFisher Scientific, A25742). All the RT-PCR experiments were repeated at least three times. The primer sequences were as follows: GAPDH, Forward: 5′- CTGGGCTACACTGAGCACC -3′ and Reverse: 5′- AAGTGGTCGTTGAGGGCAATG -3′; DAPK1, Forward: 5′- ACGTGGATGATTACTACGACACC -3′ and Reverse: 5′- TGCTTTTCTCACGGCATTTCT -3′; AKT2, Forward: 5′- ACCACAGTCATCGAGAGGACC -3′ and Reverse: 5′- GGAGCCACACTTGTAGTCCA -3′ [16, 17]. The comparative expression was calculated by the 2-ΔΔCt method, while GAPDH was set as the internal control primer.

### Western blot analysis

Total protein of samples was extracted by the ice-cold radioimmunoprecipitation lysis buffer (RIPA, Boster, AR0102), which contained protease inhibitor cocktail (Merk, P8340,1:100). We quantified the proteins through the BCA Protein Assay Kit (WSHT, EZPQ01). Subsequently, we separated the protein samples through the SDS-PAGE gels (Servicebio, G2045) and transferred them into the PVDF membranes (ABclonal, RM00018). Then, we blocked the membranes in 5% Bovine serum albumin (Boster, AR0004) and incubated them with primary antibodies: Anti-GAPDH (ABclonal, AC033, 1:1000), Anti-AKT2 antibody (ABclonal, A24009, 1:1000), and Anti-DAPK1 antibody (ABclonal, A5741, 1:1000). The membranes were then incubated by secondary antibodies: Mouse Anti-Rabbit IgG (ABclonal, AS061, 1:1000) and Goat Anti-Mouse IgG (Proteintech, SA00001, 1:1000), followed by enhanced chemiluminescence through the High Sensitivity ECL Kit (Beyotime, P0018S) to display bands.

### Statistical analysis

We analyzed differences in continuous and categorical variables between groups through the T-test and Chi-square test. Stepwise, we determined prognostic factors using both univariate and multivariate Cox's Hazards Regression methods. Survival curves were graphed using the Kaplan–Meier methods and compared using the Log-rank test. We applied the Receiver operating characteristics (ROC) curves and evaluated the prediction value of indexes through the area under the curve (AUC). All the bioinformatic statistical analyses were carried out through the R software (Version 4.2.0). For all applied tests, p-value < 0.05 was defined as statistically significant.

## Results

### Identification of anoikis-related differentially expressed genes in OV

**Fig. 2:**
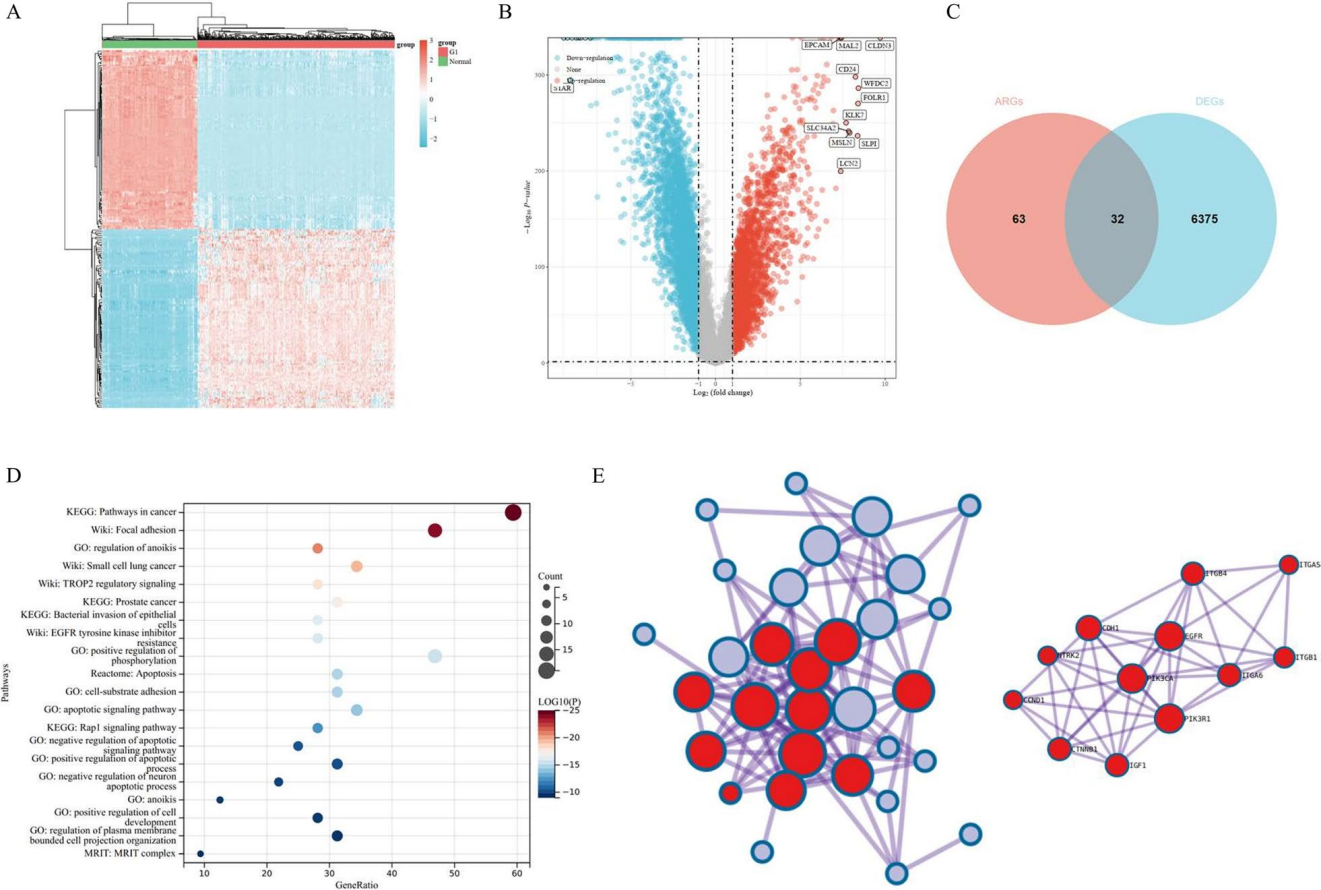
Characterization of differentially expressed anoikis-related genes (ARGs) in ovarian cancer (OV). **A** The heatmap of gene expression among normal tissues and OV tissues. The top 50 up-regulated and top 50 down-regulated genes were identified. **B** The volcano diagram showed the differentially-expressed genes (DEGs) between OV tissues and controls, among which the up-regulated and down-regulated genes were highlighted in red and blue, respectively. **C** The Venn diagram of the 32 selected DE-ARGs. **D** The top 20 Kyoto Encyclopedia of Genes and Genomes (KEGG) and Gene Ontology (GO) pathway enrichment analysis of 32 DE-ARGs. The size of the circles indicates the gene ratio, while the color represents p-value. **E** The protein–protein interaction (PPI) network diagram of the 32 DE-ARGs

Firstly, both transcriptome data and corresponding clinical features of OV patients (n = 376) were obtained from the TCGA database. As controls, we downloaded the transcriptome data of normal tissues (n = 180) from the GTEx database. In Fig. 2A, we graphed the heatmap, which could give us a brief view of gene expression profile among normal tissues and OV tissues. Stepwise, in Fig. 2B, we graphed the volcano diagram, which showed the differentially-expressed genes (DEGs) between OV tissues and controls. Based on Fig. 2A and B, we identified 6406 DEGs, among which 2333 genes were up-regulated, while 4073 genes were down-regulated in OV tissues, compared with normal controls. Based on the Genecards dataset, we obtained 95 ARGs (Relevance Score ≥ 2). Stepwise, we filtered 32 ARGs, which were differentially expressed between normal controls and OV tissues according to the Venn diagram (Fig. 2C). Then, we carried out GO and KEGG pathway enrichment analysis of the 32 potential DE-ARGs via the Metascape website (https://metascape.org) [19]. The top 20 most significant pathways were listed, which were mainly enriched in pathways in cancer, regulation of anoikis, and focal adhesion, etc. (Fig. 2D). In order to provide hints for protein interactions network of 32 filtered DE-ARGs, in Fig. 2E, we conducted the protein–protein interaction (PPI) analysis through Metascape (https://metascape.org/), a database that could provide screens for a PPI network. The analysis revealed that these 32 genes might interact with each other, especially the hub genes (in red) including EGFR, PIK3CA, and PIK3R1, etc. (Fig. 2E, right).

### Establishment and estimation of the prognostic signature based on the anoikis-related genes

**Fig. 3:**
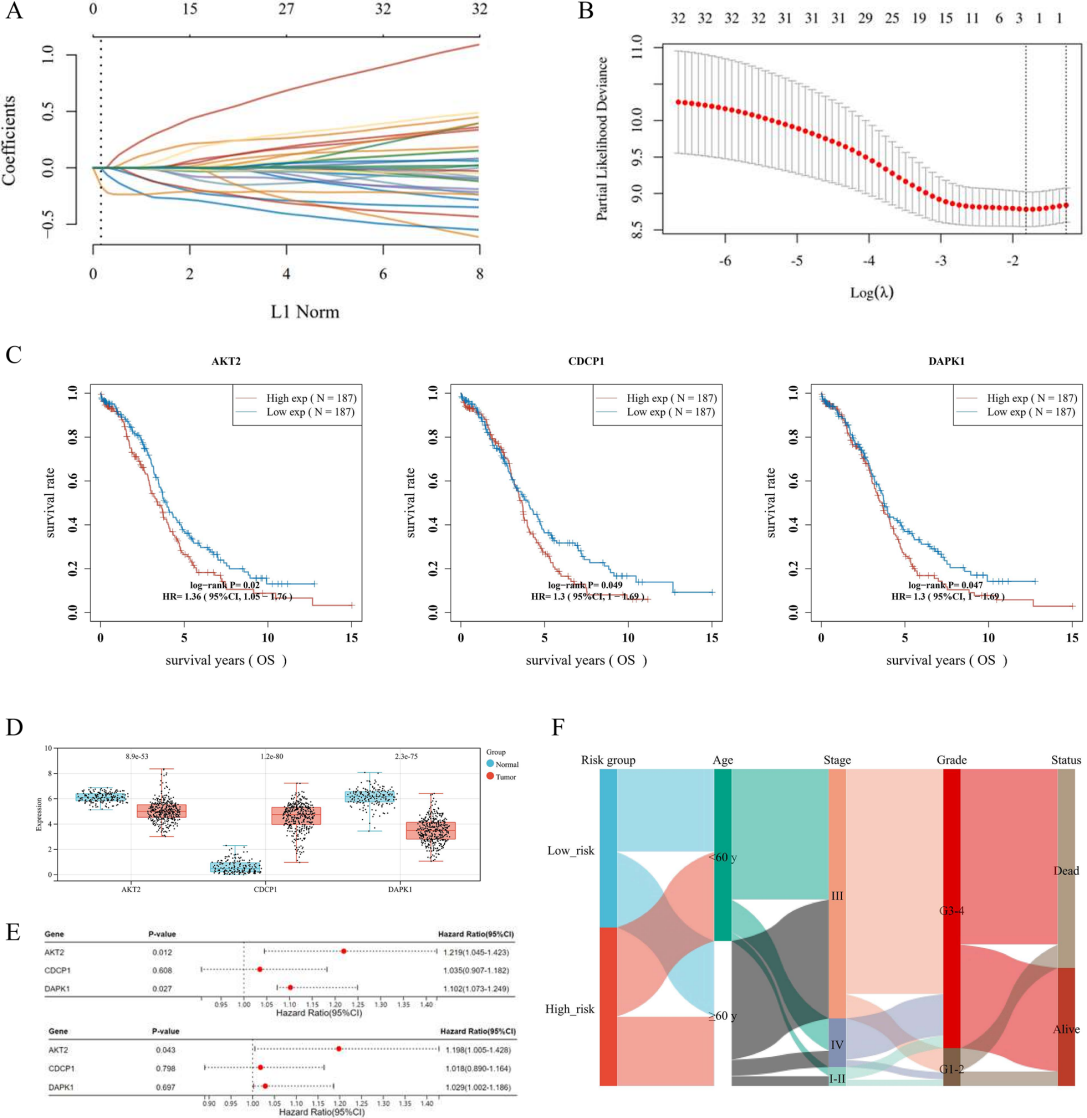
Establishment of ovarian cancer (OV) prognostic signature based on the anoikis-related genes (ARGs). **A**, **B** The λ selection diagram of LASSO parameter selection with tenfold cross-validation. **C** The Kaplan–Meier (K-M) survival curves of the optimal prognostic ARGs, including AKT2, CDCP1, and DAPK1. **D** The expression distribution of the 3 potential prognostic ARGs in OV tissues and normal controls. **E** The forest plot represented the prognostic ability of the 3 optimal prognostic ARGs, evaluated through the LASSO-Cox analysis. **F**, **C** The Sankey diagram for the anoikis-related subtypes and clinical features, including age, FIGO stage, grade, and survival status

Through the LASSO analysis, we selected 3 potential prognostic genes (including AKT2, CDCP1, and DAPK1) from the 32 DE-ARGs (Fig. 3A, B). As shown in the K-M survival curves (Fig. 3C), OV patients with high expression of AKT2, CDCP1, and DAPK1 suffered worse OS. The expression distributions of the 3 prognostic ARGs in OV tissues and normal controls were presented in Fig. 3D. To enhance model explicability, we conducted both univariate and multivariate Cox Regression analysis to distinguish prognostic genes for the anoikis-related signature, namely AKT2, and DAPK1 (Fig. 3E). Ultimately, we constructed an anoikis-related 2-gene prognostic signature as follows: Risk score = (0.0932) * AKT2 + (0.0121) * DAPK1. The Sankey diagram indicated the association between the anoikis-related subtypes and clinical features, including age, FIGO stage, grade, and survival status (Fig. 3F).

**Fig. 4:**
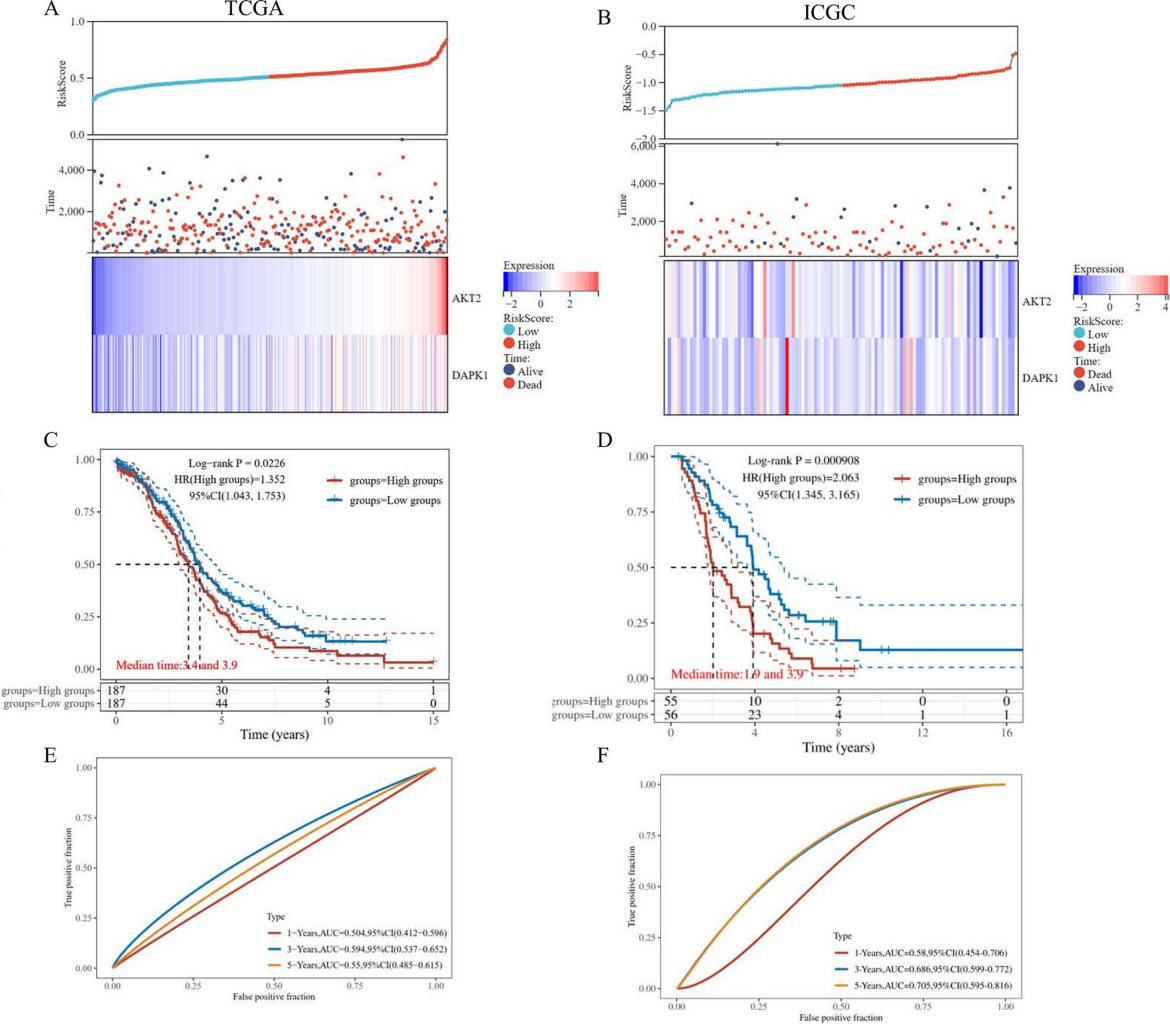
Validation of the anoikis-related prognostic signature. The distribution and scatter diagrams (top) represented the Risk score of each OV patient, referring to corresponding survival time (days), and survival status, in the TCGA-OV training cohort (**A**) and ICGC-OV validation cohort (**B**). The heatmaps (bottom) showed gene expression of the 2-gene signature (AKT2 and DAPK1) between low-risk and high-risk groups. The overall survival (OS) Kaplan–Meier (K-M) curves for patients in the TCGA-OV (**C**) and ICGC-OV cohort (**D**), which were classified into the low-risk and high-risk groups based on the anoikis-related prognostic signature. The ROC analysis of the anoikis-related prognostic signature among the TCGA-OV training cohort (**E**) and ICGC-OV validation cohort (**F**)

Based on the above formula, we calculated the Risk score of every OV patient, in the TCGA-OV training cohort (n = 374) and ICGC-OV validation cohort (n = 111). Next, we divided OV patients into low-risk and high-risk groups, referring to the median as the cut-off value. In Fig. 3A and B (top and middle), we showed the distribution profiles of Risk scores in both training cohort and validation cohorts, referring to corresponding survival time and status. The results also demonstrated that AKT2 and DAPK1 were highly expressed in the high-risk group (Fig. 4A and B, bottom). Through the K-M curves, we found that patients in the high-risk group suffered worse OS in both the training cohort (p-value = 0.0226) and validation cohort (p-value = 0.0009) (Fig. 4C and D). The time-dependent ROC analysis indicated that the anoikis-related 2-gene signature had promising prognostic values for 1-year, 3-year, and 5-year OS prediction in both cohorts (Fig. 4E and F).

### Single-cell analysis and somatic alteration landscape of the anoikis-related signature

**Fig. 5:**
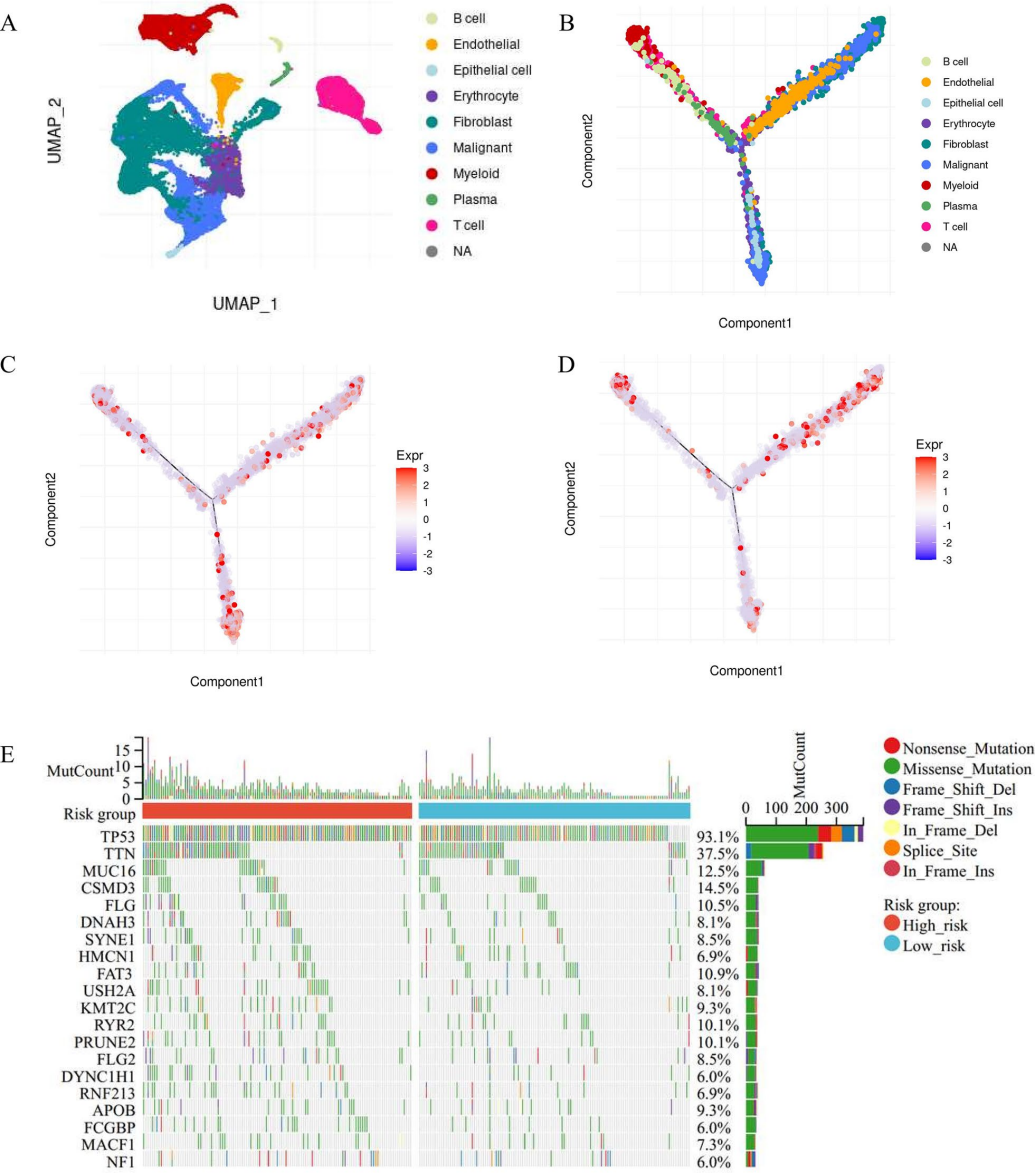
The single-cell analysis and somatic alteration landscape of the anoikis-related signature. **A** The UMAP diagram showed high-quality cells from a 10 × Genomics dataset from Qian and colleagues [18]. Ten typical cell types were defined by specific markers. Each dot represented an individual cell colored as annotated. **B** The pseudo time trajectory analysis of 10 cell types from OV tissues. Each color represented specific cell types. The pseudo time trajectory analysis of cells in OV tissues with **C** AKT2 and **D** DAPK1 expression. **E** The genomic aberrations landscape of the genes in the two anoikis-related clusters of TCGA-OV patients. The frequency of alterations in the top 20 genes was shown

Based on the single-cell dataset from Qian and colleagues [18], which contained 5 OV patients and 2 controls, we evaluated 10 typical cell types defined by specific markers (Fig. 5A). The pseudo-time trajectory analysis indicated the potential evolutionary relationships between different types of cells, during the cell state transition (Fig. 5B). The trajectory inference revealed the evolutionary pathways of T cells, B cells, and Myeloid cells from OV lesions, with (Fig. 5C) AKT2 and (Fig. 5D) DAPK1 expression, which overlapped along pseudotime. The single-cell analysis was conducted through the TIGER website (http://tiger.canceromics.org/).

To reveal the genomic landscape of this inter-lesion diversity according to the anoikis-related signature, we evaluated the Somatic cell copy number alternation (SCNA) and mutation frequency among the TCGA-OV cohort. The genes with the highest mutation frequency are TP53 (93.1%), TTN (37.5%), and MUC16 (12.5%) (Fig. 5E).

### Construction and validation of the anoikis-associated nomogram

**Fig. 6:**
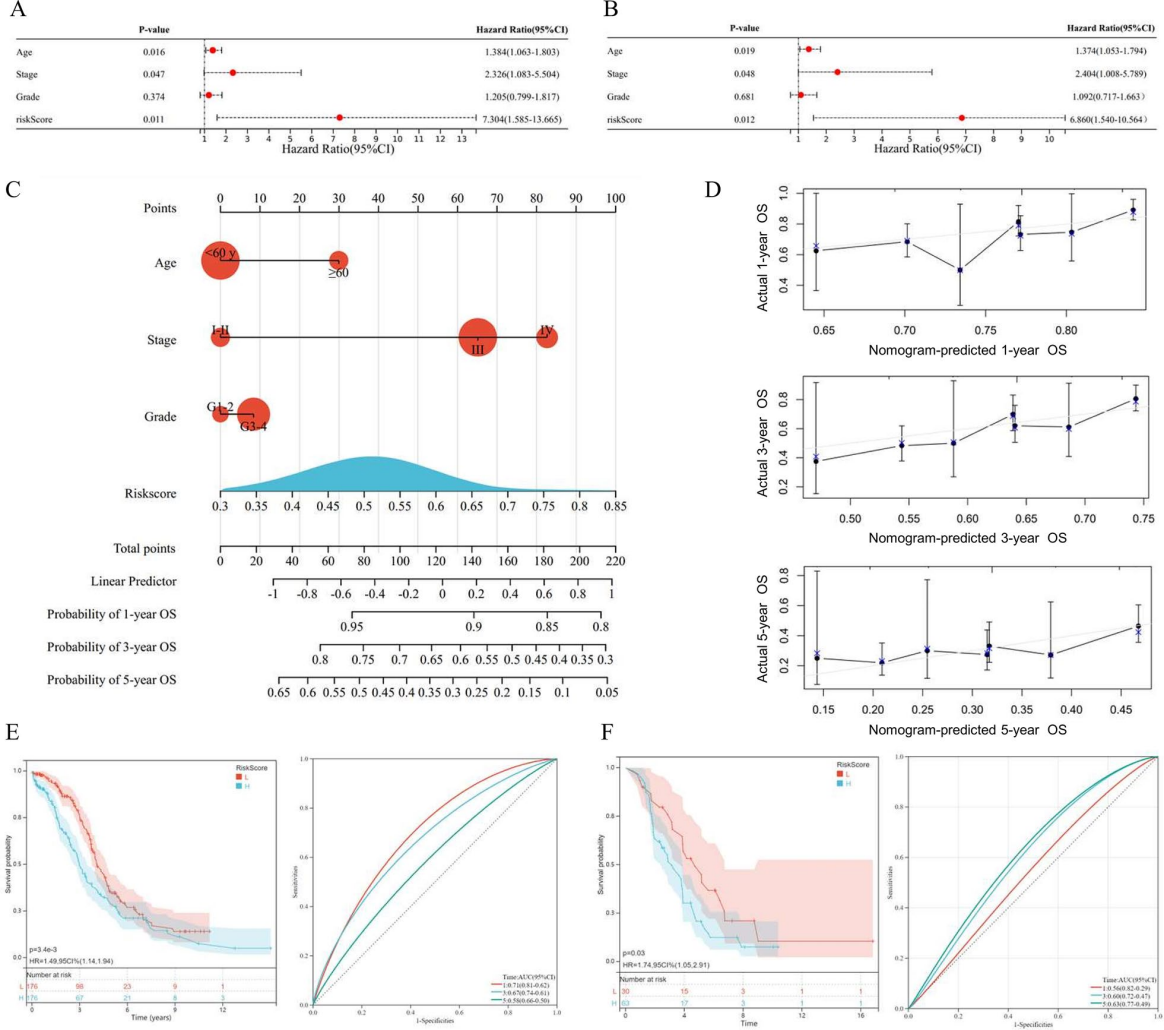
Construction and validation of the anoikis-related nomogram for ovarian cancer (OV). The forest plots for univariate (**A**) and multivariate (**B**) Cox Regression analysis for overall survival (OS), based on the anoikis-related 2-gene signature and clinical features, including age, clinical FIGO stage, and pathological grade. **C** The prognostic nomogram model for 1-year, 3-year, and 5-year OS among OV patients, based on the anoikis-related signature and clinical features selected by the univariate and multivariate Cox Regression analysis. **D** The calibration plots of the anoikis-related nomogram for predicting 1-year, 3-year, and 5-year OS (top, middle, and bottom, respectively). The Kaplan–Meier (K-M) curves for patients classified by the anoikis-related nomogram score, in the TCGA-OV training cohort (**E**) and ICGC-OV validation cohort (**F**)

Based on the anoikis-related 2-gene signature and clinical features, including age, clinical FIGO stage, and pathological grade, we conducted both univariate (Fig. 6A) and multivariate (Fig. 6B) Cox Regression analyses to determine the independent prognostic indicators for OV. The results demonstrated that age (p-value = 0.019) and FIGO stage (p-value = 0.048) were prognostic factors for OS, besides Riskscore (p-value = 0.012). According to the selected prognostic indicators, we constructed a quantitative nomogram for 1-year, 3-year, and 5-year OS prediction, with a C-index of 0.6004 (Fig. 6C). In Fig. 6D, the calibration plots indicated ideal consistency between predicted and observed 1-year, 3-year, and 5-year survival (top, middle, and bottom, respectively) among OV patients. Furthermore, we validated the optimum performance of the nomogram in both the TCGA-OV training cohort (Fig. 6E, p-value < 0.001) and the ICGC-OV validation cohort (Fig. 6F, p-value = 0.030), through the K-M curve analysis and time-dependent ROC analysis.

### The immune microenvironment landscape related to the anoikis-related signature

**Fig. 7:**
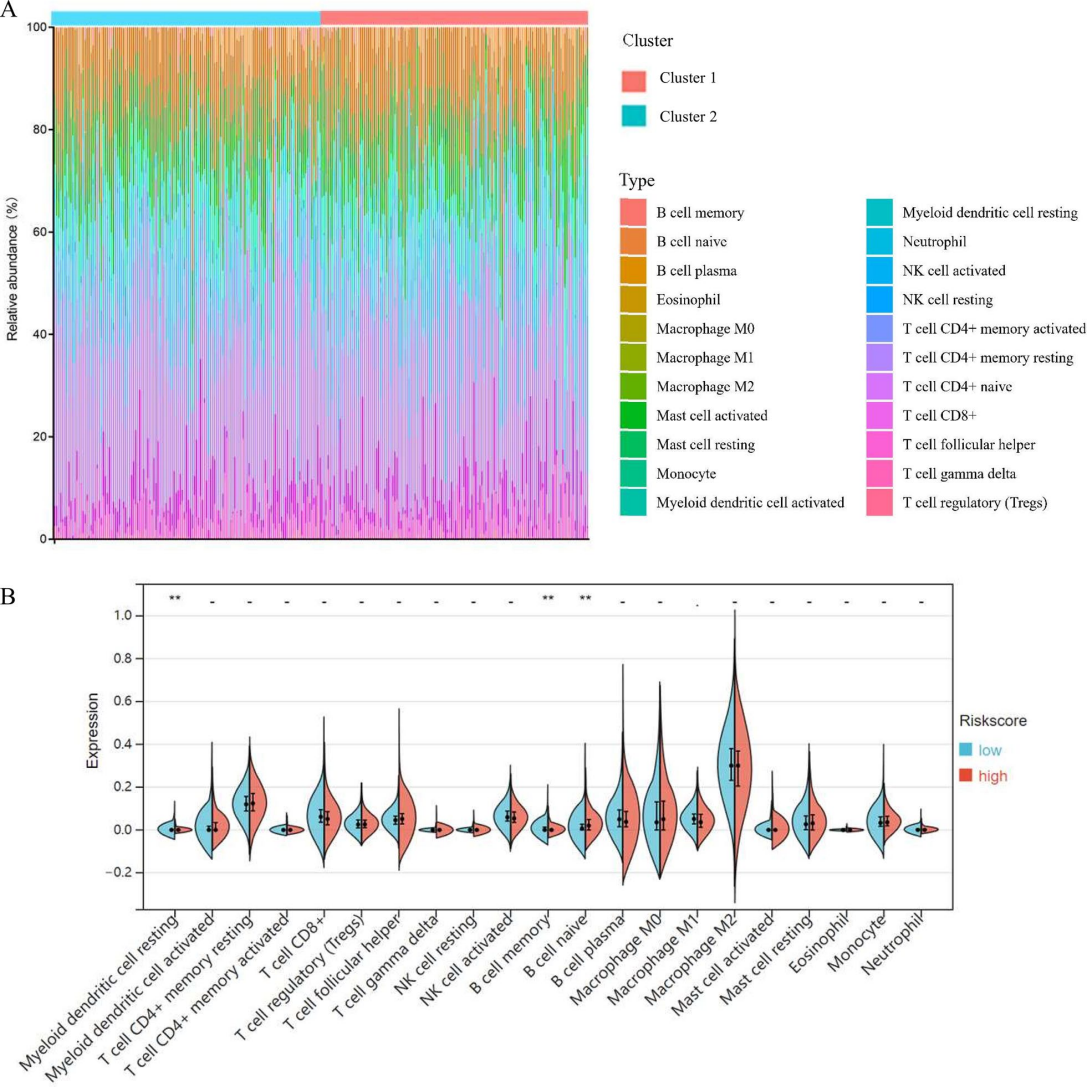
The analysis for the tumor immune microenvironment related to the anoikis-related signature. **A** The Boxplots showed the composition of 22 typical immune cells infiltrating the TCGA-OV samples, which were evaluated via the anoikis-related signature. OV patients were classified into low-risk and high-risk groups by the anoikis-related signature. **B** The Violin diagrams indicated the expression of the 22 immune cells infiltration between two risk groups. **p-value < 0.01

Growing evidence claimed that through the cross-talk between immune cells and tumor cells, the tumor immune microenvironment could largely contribute to the OV progression procedure [19]. Accordingly, to determine the association between the anoikis and immune infiltration landscape in OV, we conducted the CIBERSORT analysis to evaluate the immune microenvironment, which was stratified by the anoikis-related signature. In Fig. 7A, we summarized the composition of the 22 typical immune cells infiltrating tumor tissues, among both the low-risk and high-risk TCGA-OV cohorts. According to the CIBERSORT algorithm, 3 out of the 22 typical immune cells, including resting Myeloid Dendritic Cells (DCs), memory B cells, and naïve B cells were significantly upregulated in the high-risk group, compared to the low-risk group (Fig. 7B, p-value < 0.01).

### Assessment of immunotherapy and chemotherapy response related to the anoikis-related signature

**Fig. 8:**
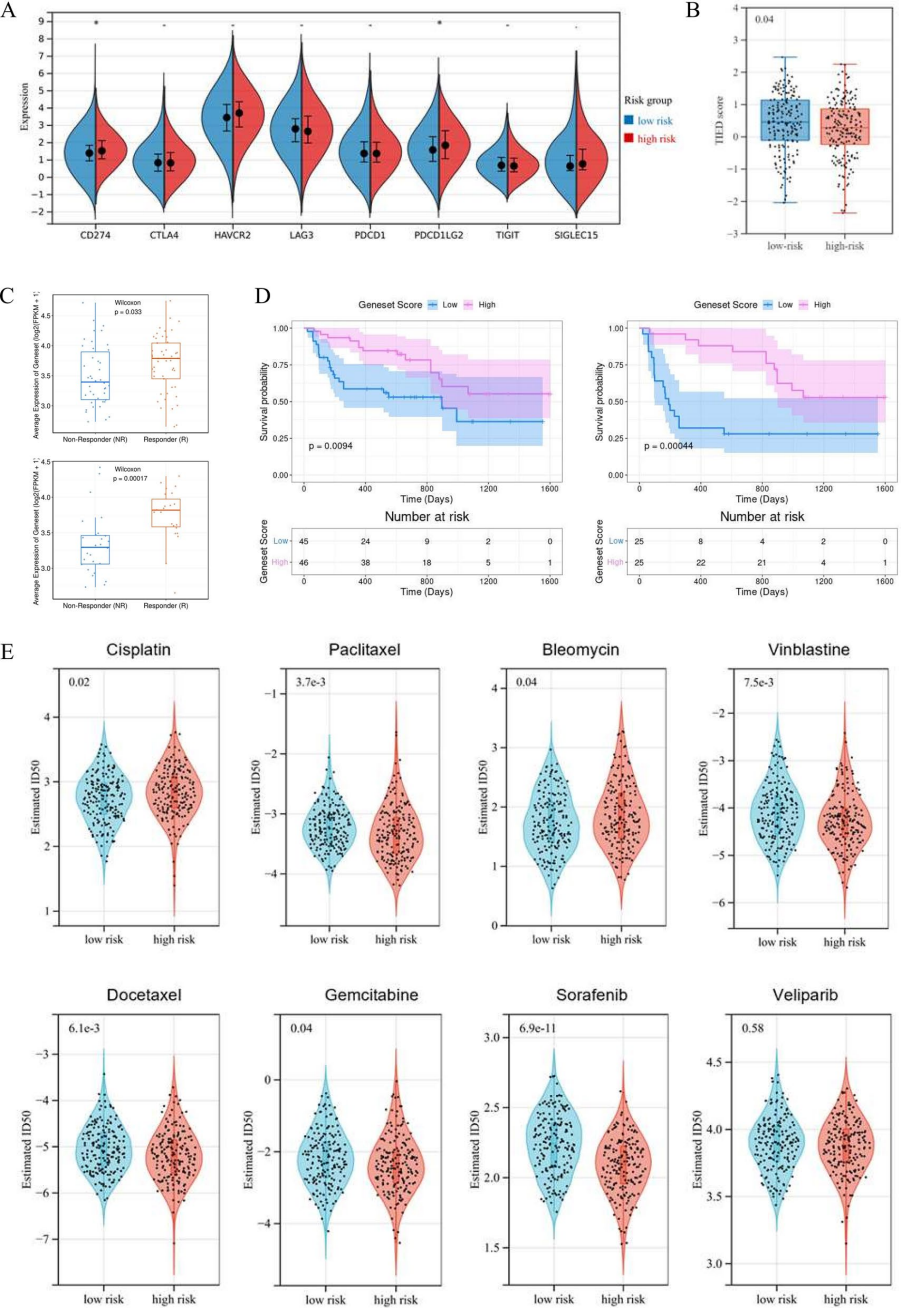
Assessment of immunotherapy and chemotherapy response related to the anoikis-related signature among OV patients. **A** The boxplots of the gene expression distribution for 8 typical immune checkpoints, including CTLA4, CD274, LAG3, HAVCR2, SIGLEC15, PDCD1, PDCD1LG2, and TIGIT, between the two risk groups classified by the anoikis-related signature. **B** The prediction of patient response to immune checkpoint blockade (ICB) therapies, according to the Tumor Immune Dysfunction and Exclusion (TIDE) score. **C** Based on the PRJEB23709 dataset [20], we compared the distribution of Riskscores among responders and non-responders to anti-CTLA-4 + anti-PD-1 (top) and anti-PD-1 (bottom) immunotherapies. (D) The Kaplan–Meier (K-M) curves for patients classified by the anoikis-related signature, in anti-CTLA-4 + anti-PD-1 (left) and anti-PD-1 (right) cohorts. (E) The violin diagrams of estimated half maximal inhibitory concentration (IC50) values among OV patients, in terms of 8 typical chemotherapies (including Bleomycin, Docetaxel, Cisplatin, Paclitaxel, Gemcitabine, Sorafenib, Veliparib, and Vinblastine). The chemotherapy sensitivity was estimated according to the Genomics of Drug Sensitivity in Cancer (GDSC) database. *P-value < 0.05

Additionally, we evaluated the association between the anoikis-related gene signature and immunotherapy sensitivity. Typical immune checkpoints, including CTLA4, CD274, LAG3, HAVCR2, SIGLEC15, PDCD1, PDCD1LG2, and TIGIT were selected to be immune-checkpoint–relevant transcripts and the expression values of these eight genes were extracted. Based on the anoikis-related riskscore formula defined, we stratified TCGA-OV patients into two risk groups. Next, we evaluated the expression of these typical immune checkpoint molecules among anoikis-related risk groups. The result implied that CD274 and PDCD1LG2 were up-regulated among the high-risk group (p-value < 0.05, Fig. 8A). Accordingly, patients with higher anoikis-related Riskscore were more likely to benefit from immunotherapies focus on these 2 immune checkpoints. Through the Tumor Immune Dysfunction and Exclusion (TIDE) algorithm, we also predicted OV patient response to immune checkpoint blockade (ICB) therapies. The results indicated that low-risk OV patients had higher TIDE scores, which represented poorer survival after ICB therapies (p-value < 0.05, Fig. 8B). Moreover, based on the PRJEB23709 dataset [20], we evaluated the prediction value of the anoikis-related signature among tumor patients treated with immunotherapy. Figure 8C demonstrated that responders had higher Riskscore compared with non-responders, among patients who received anti-CTLA-4 + anti-PD-1 and anti-PD-1 therapies. Through the K-M survival analysis, we found that the high-risk patients were more likely to benefit from immunotherapy, including both anti-CTLA-4 and anti-PD-1 treatment (p-value < 0.05, Fig. 8D).

Based on the Genomics of Drug Sensitivity in Cancer (GDSC) dataset, we compared the estimated half maximal inhibitory concentration (IC50) values of 8 typical OV chemotherapies between two risk groups. The results implied that the estimated IC50 values of Cisplatin and Bleomycin in the high-risk group were higher, while the IC50 values of Paclitaxel, Vinblastine, Docetaxel, Gemcitabine, and Sorafenib were significantly higher in the low-risk group (p-value < 0.05, Fig. 8E).

### Pan-cancer analysis of the anoikis-associated signature

**Fig. 9:**
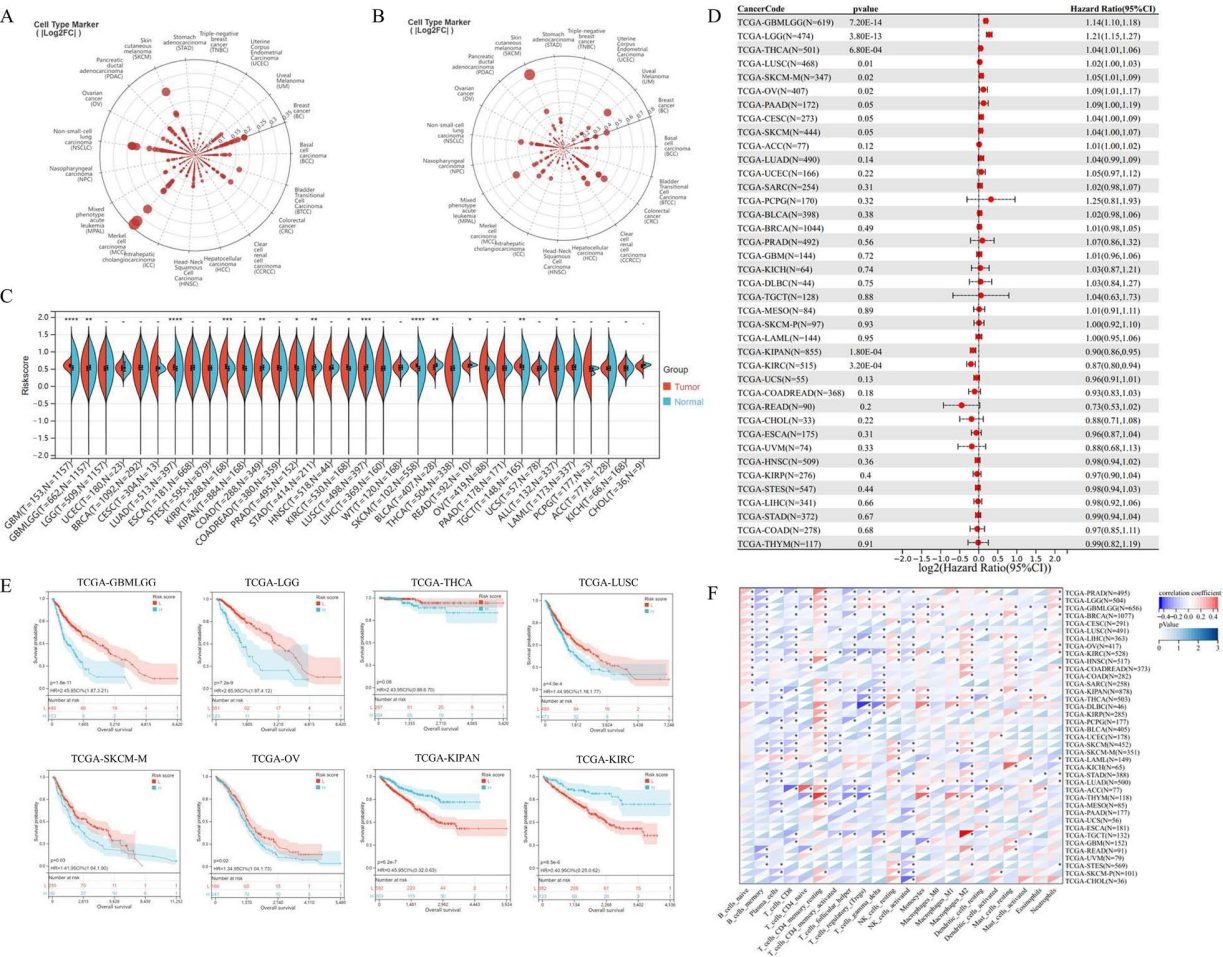
Pan-cancer analysis of the anoikis-related signature. The Radar chart represented the gene expression of AKT2 (**A**) and DAPK1 (**B**) in pan-cancer. **C** The violin plots presented the anoikis-related Riskscore level of tumor tissues and normal controls in pan-cancer. **D** The forest plot to distinguish the prognostic value of the anoikis-related prognostic signature in pan-cancer through the Cox Regression algorithm. **E** The Kaplan–Meier (K-M) survival curves for Glioma (GBMLGG), Low-grade glioma (LGG), Thyroid cancer (THCA), Lung squamous cell carcinoma (LUSC), Skin cutaneous melanoma (SKCM), Ovarian serous cystadenocarcinoma (OV), Pan-kidney (KIPAN), and Kidney clear renal cell carcinoma (KIRC) in the TCGA cohorts, which were stratified by the anoikis-related signature. **F** The relationship between anoikis-related Riskscore and immune cell infiltration in pan-cancer, which was analyzed BY the CIBERSORT algorithm

In order to evaluate the application of the anoikis-associated signature in cancers, we conducted a pan-cancer analysis of the 34 tumors in the TCGA dataset. Firstly, we compared the gene expression of AKT2 (Fig. 9A) and DAPK1 (Fig. 9B) in pan-cancer, among which Merkel cell carcinoma and Skin cutaneous melanoma (SKCM) ranked the highest AKT2 and DAPK1 expression, respectively. In Fig. 9C, we compared the Riskscores of OV tissues and normal controls. The results indicated that almost all cancers had different Riskscore levels, including OV. The prognostic value of the anoikis-associated signature was also analyzed in the TCGA pan-cancer cohorts through the Cox Regression algorithm (Fig. 9D). The Kaplan–Meier (K-M) survival curves validated the optimum performance of the signature (p-value < 0.05) in Glioma (GBMLGG), Low-grade glioma (LGG), Thyroid cancer (THCA), Lung squamous cell carcinoma (LUSC), Skin cutaneous melanoma (SKCM), Ovarian serous cystadenocarcinoma (OV), Pan-kidney (KIPAN), and Kidney clear renal cell carcinoma (KIRC) in the TCGA cohorts, which were stratified by the anoikis-related signature (Fig. 9E). Furthermore, we explored the relationship between anoikis-related Riskscore and immune cell infiltration in pan-cancer (Fig. 9F). The results suggested that memory CD4 + T cells and Macrophage M2 cells were positively related to the anoikis-related signature in pan-cancer, while Treg cells were inversely related.

### Aberrant upregulation of DAPK1 was related to metastasis and poor prognosis in OV

**Fig. 10:**
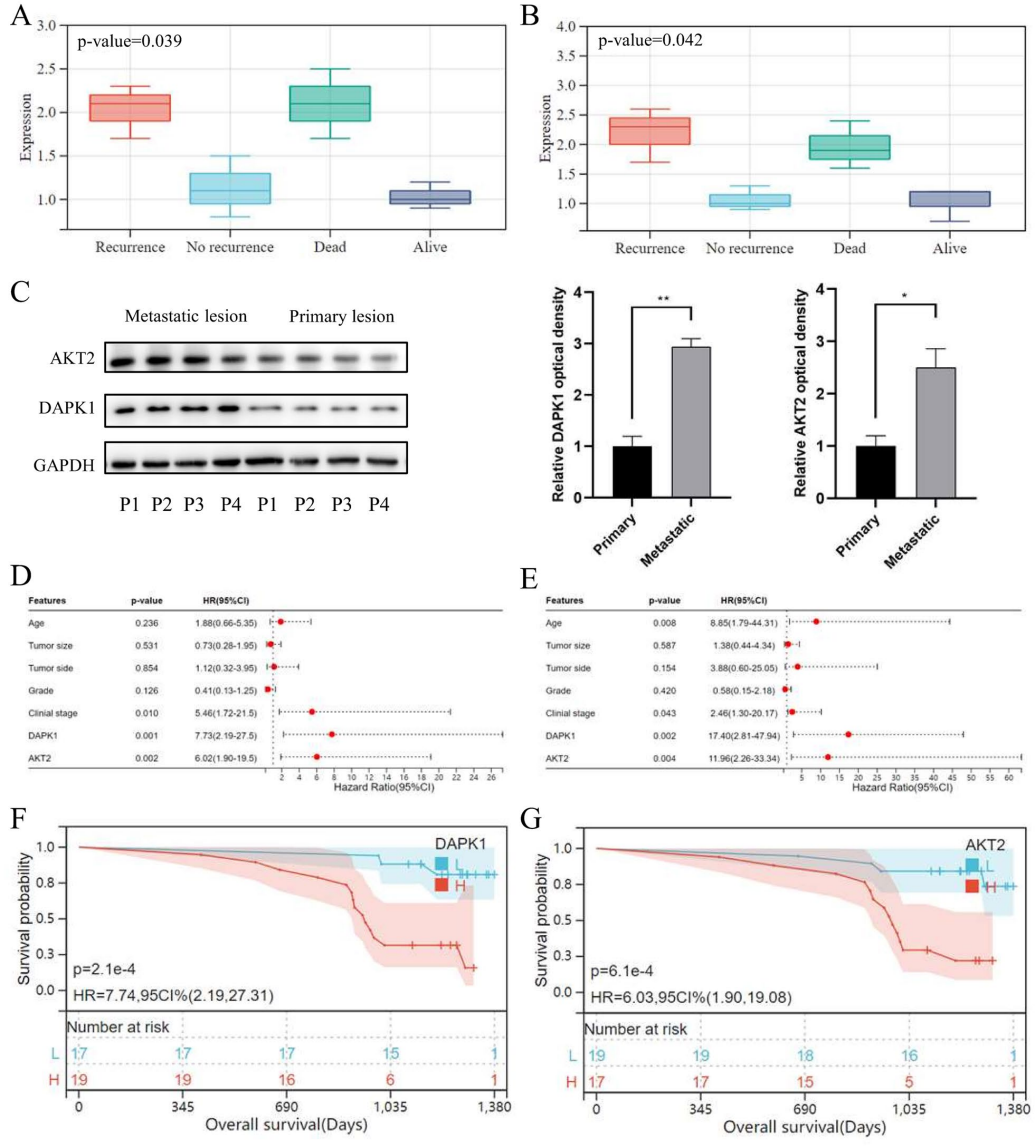
The anoikis-related genes (DAPK1 and AKT2) could predict prognosis of ovarian cancer (OV) patients. The gene expression of **A** DAPK1 and **B** AKT2 in OV tissues, which was measured by the qRT-PCR analysis. **C** The Western blotting analysis for DAPK1 and AKT2 expression in both primary and metastatic OV lesions from 4 representative patients. The forest plot of **D** univariate and **E** multivariate Cox regression analysis of OV patient survival, according to clinical features and anoikis-related genes (DAPK1 and AKT2). The Kaplan–Meier (K-M) survival curves for the overall survival (OS) of OV patients, which were stratified by the expression of **F** DAPK1 and **G** AKT2

We involved 36 OV patients at our institution, who were followed-up for 37.88 (31.48–42.72) months. The clinical features of the involved OV patients were showed in Additional file 1: Table S1. We measured the RNA expression of DAPK1 and AKT2 in OV tissues through qRT-PCR analysis. The result proved that OV patients suffered poor prognosis had higher DAPK1 and AKT2 expressions (p-value = 0.04, Fig. 10A, B). We also conducted the Western Blotting, which further revealed that protein expression of DAPK1 and AKT2 was significantly increased in metastatic lesions (Fig. 10C). Stepwise, we applied both univariate (Fig. 10D) and multivariate (Fig. 10E) Cox regression analyses for prognostic clinical features, which indicated that DAPK1 (p-value = 0.002) and AKT2 (p-value = 0.004) were prognostic factors, in addition to age (p-value = 0.008) and clinical FIGO stage (p-value = 0.043). The Kaplan–Meier (K-M) survival curves for the overall survival (OS) revealed that OV patients with higher DAPK1 and AKT2 expression suffered worse prognosis (p-value < 0.01, Fig. 10F, G). These findings were consisted with the results of bioinformatics analysis.

**Fig. 11:**
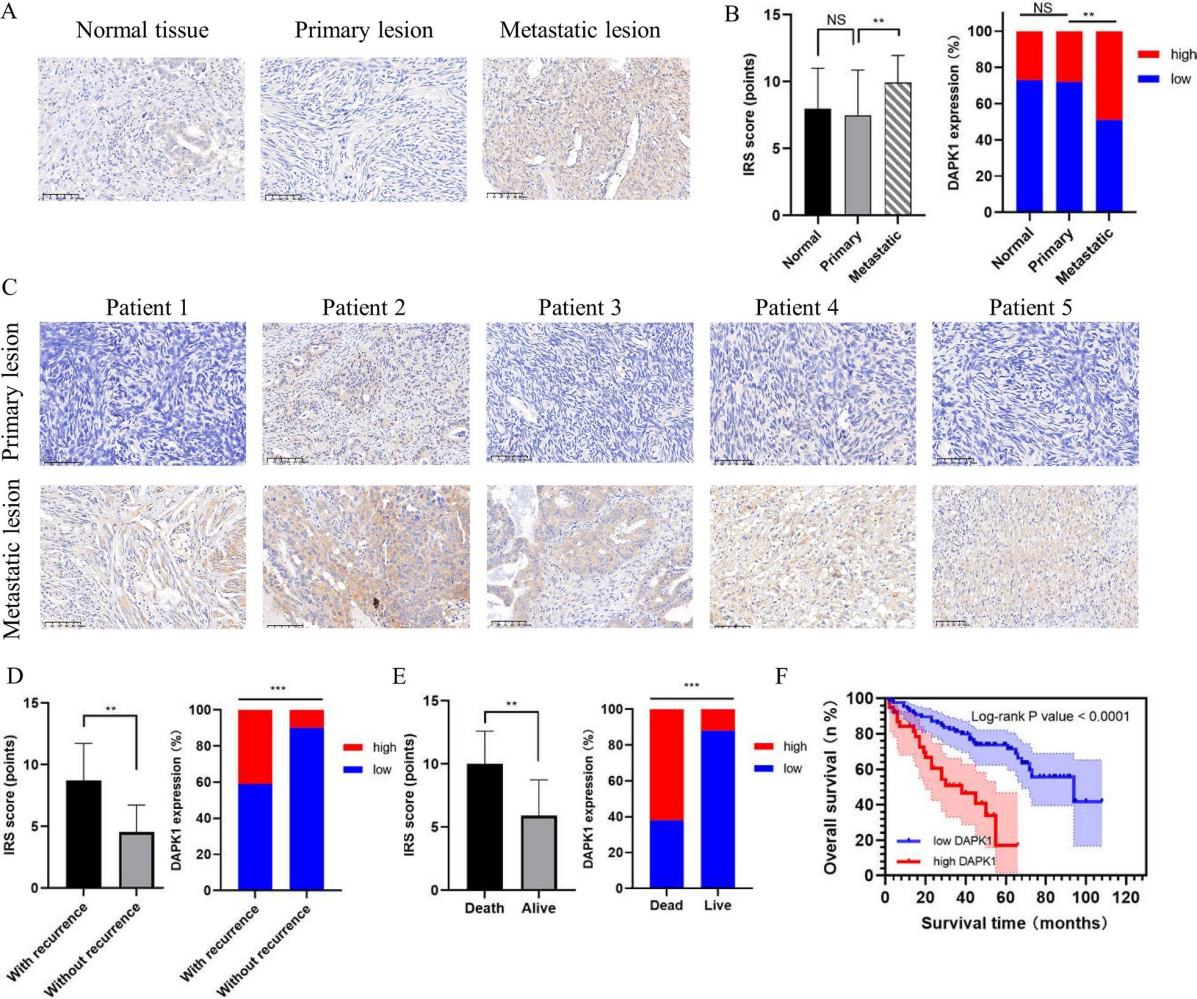
DAPK1 expression up-regulated in OV metastatic lesions and was related to poor survival. **A** The representative immunohistochemistry (IHC) staining images (original magnification × 200) for DAPK1 expression in normal ovary tissue, primary OV lesions, and metastatic OV lesions were listed. **B** Metastatic OV lesions had higher DAPK1 expression, compared with normal controls and primary OV lesions, which were measured by the IRS score. **C** The IHC staining images (original magnification × 200) for primary and metastatic lesions from representative OV patients. The DAPK1 expression was up-regulated in OV patients who suffered (**D**) recurrence or (**E**) death, which was analyzed through IHC staining. **F** Kaplan–Meier (K-M) curves for the overall survival (OS) among 125 OV patients, which were stratified based on DAPK1 expression

However, as for DAPK1, there were no research focused on the anoikis pattern in OV metastasis till now. Thus, we mainly focused on the relationship between DAPK1 expression an OV metastasis in our study. The IHC analysis of tissue microarrays showed that DAPK1 expression staining was located at both cytosol and nucleus of tumor cells (Fig. 11A). Compared with primary OV lesions (IRS score = 7.49 ± 3.38) and normal controls (IRS score = 8.00 ± 3.01), metastatic lesions had significantly higher DAPK1 expression (IRS score = 9.95 ± 2.03) (Fig. 11B). In Fig. 11C, we listed 5 representative pairs of IHC staining images for primary and metastatic tumor lesions. Through the IHC staining analysis of tissue microarrays based on 125 OV individuals, we found that DAPK1 expression significantly increased among OV patients who suffered recurrence or death (Fig. 11D and E, p-value < 0.05). In Table 1, we listed the relationship between DAPK1 expression and clinicopathological characteristics, among which only the FIGO stage had significant association with DAPK1 expression (p-value = 0.002). The median OS of OV patients were 34 months (range 14–52). In Fig. 10F, among OV patients, the K-M survival curves revealed that DAPK1 expression was related to OS (p-value < 0.0001). In Table 2, we conducted both univariate and multivariate analyses to identify independent prognostic factors, including the FIGO stage (HR 3.873; 95% CI, 1.465–10.239; p-value = 0.006), DAPK1 expression (HR 5.196; 95% CI, 2.789–9.681; p-value = 0.001). Collectively, DAPK1, one of the key ARGs, was significantly related to OV metastasis and poor prognosis.

**Table 1:** The correlation between DAPK1 expression and clinicopathological features of 125 OV patients

Characteristic	No. of patients	DAPK1 expression		p-value
		Low (IRS score < 6)	High (IRS score ≥ 6)	
Age (n, %)				0.855
< 55 years	56(44.8%)	39(31.2%)	17(13.6%)	–
≥ 55 years	69(55.2%)	47(37.6%)	22(17.6%)	–
FIGO stage (n, %)				0.002
I–II	45(36.0%)	39(31.2%)	6(4.8%)	–
III–IV	80(64.0%)	47(37.6%)	33(26.4%)	–
Pathology stage (n, %)				0.953
I–II	54(43.2%)	37(29.6%)	17(13.6%)	–
III	71(56.8%)	49(39.2%)	22(17.6%)	–
Histology type (n, %)				0.331
Serous	78(62.4%)	53(42.4%)	25(20.0%)	–
Mucous	11(8.8%)	10(8.0%)	1(0.8%)	–
Endometrioid	14(11.2%)	8(6.4%)	6(4.8%)	–
Other types	22(17.6%)	15(12.0%)	7(5.6%)	–
Tumor diameter (n, %)				0.604
< 10 cm	62(49.6%)	44(35.2%)	18(14.4%)	–
≥ 10 cm	63(50.4%)	42(33.6%)	21(16.8%)	–
Serum CA125 (n, %)				0.051
< 35 U/ml	20(16.0%)	18(14.4%)	2(1.6%)	–
≥ 35 U/ml	105(84.0%)	68(54.4%)	37(29.6%)	–

*FIGO stage* Federation International of Gynecology and Obstetrics stage

**Table 2:** Univariate and multivariate Cox Regression analysis of prognostic factors of the 125 OV patients

Characteristic	Univariate Analysis		Multivariate Analysis	
	HR (95% CI)	P-value	HR (95% CI)	P-value
Age				
< 55 years	Reference	–	Reference	–
≥ 55 years	1.052(0.595–1.862)	0.861	1.189(0.652–2.167)	0.573
FIGO stage				
I–II	Reference	–	Reference	–
III–IV	4.238(1.897–9.471)	0.001	3.873(1.465–10.239)	0.006
Pathology stage				
I–II	Reference	–	Reference	–
III	0.727(0.413–1.282)	0.271	0.731(0.373–1.431)	0.360
Histology type		0.850		0.589
Serous	Reference	–	Reference	–
Mucous	0.591(0.181–1.932)	0.384	1.902(0.390–9.286)	0.427
Endometrioid	0.875(0.341–2.245)	0.780	0.910(0.330–2.510)	0.855
Other types	0.977(0.450–2.122)	0.953	1.676(0.679–4.138)	0.263
Tumor diameter				
< 10 cm	Reference	–	Reference	–
≥ 10 cm	1.253(0.710–2.211)	0.437	1.483(0.796–2.764)	0.215
Serum CA125				
< 35 U/ml	Reference	–	Reference	–
≥ 35 U/ml	2.680(0.960–7.486)	0.060	1.202(0.304–4.754)	0.793
DAPK1 expression				
Low (IRS score < 6)	Reference	–	Reference	–
High (IRS score ≥ 6)	5.994(3.290–10.921)	0.001	5.196(2.789–9.681)	0.001

*FIGO stage* Federation of International of Gynecologists and Obstetricians stage, *HR* hazard ratio, *95% CI* 95% confidence interval

## Discussion

OV was one of the most lethal gynecological malignancies worldwide, mainly due to the high recurrence rate and lack of specific symptoms [1, 3]. Therefore, identifying reliable prognostic biomarkers and exploring the underlying mechanism is of great urgency for personalized OV treatment. Recently, emerging evidence supported that anoikis, a programmed cell death induced by cell detachment from ECM, played a crucial role in tumor metastasis [5]. Therefore, in our research, we comprehensively evaluated and validated an anoikis-related signature (including AKT2 and DAPK1), which was associated with tumor immune microenvironment and sensitivity to immunotherapy/ chemotherapy. Moreover, we aimed to explore the vital role of ARGs, especially DAPK1, in OV metastasis, in order to guide clinical decision-making and present a new therapeutic target for OV patients.

Up till now, though several studies have focused on the association between anoikis and OV metastasis, none anoikis-related signature has been applied to clinical practice yet, partly owing to the limitations in specificity and sensitivity. Recently, Qian and colleagues created a prognostic signature including 5 ARGs (STAT1, SNAI1, RB1, SFRP1, and AKT2) [21], all of which had been reported to be related to anoikis. However, the signature had limited prognostic value in validation cohorts, especially the GSE30161 cohort (HR 1.147; 95%CI 0.448–2.938; p-value = 0.775). Accordingly, we tried to distinguish a promising anoikis-related signature from 95 potential ARGs downloaded from the Genecards database (Relevance Score ≥ 2). We identified a 2-gene signature (including AKT2 and DAPK1), which was validated in both the TCGA-OV training cohort (p-value < 0.001) and the ICGC-OV validation cohort (p-value = 0.030). To the best of our knowledge, our research is initial to define the anoikis-related signature of AKT2 and DAPK1, which had satisfactory prognostic value for OV patients.

AKT2, a homolog of AKT1, could encode a serine/threonine protein kinase which is largely amplified in pancreatic and ovarian cancers [22]. Moro and colleagues reported that depletion of mtDNA could prevent anoikis and promote migration onto the basement membrane by upregulation of p85 and p110 PI3K subunits, which could result in AKT2 activation and phosphorylation of downstream substrates in prostate cancer [23]. The research team of Fujio claimed that AKT2 could function as an anti-anoikis gene during the cellular differentiation procedure, a property that might contribute to oncogenicity [24]. In OV, Zheng and colleagues concluded that AKT2 could contribute to promote OV migration and invasion via the AKT2-PKM2-STAT3/NF-κB axis [25]. As for DAPK1, Qiu et al. demonstrated that DAPK1, a tumor suppressor that could activate cell death, was directly linked to anoikis activation via rigidity sensing [26]. In non-small cell lung cancer, Phosphorylation of DAPK at Ser734 by ERK was essential for p53 transcriptional activity, which was required for anoikis [27]. However, there were relatively few studies focused on the anoikis pattern in OV, especially for DAPK1, so future exploration of the underlying mechanisms is critical.

Nowadays, growing evidence supported the cross-talk between immune cells and tumor cells, along with increasing breakthroughs in the realm of immune checkpoint inhibitors [19]. In order to investigate the association between anoikis and the immune infiltration landscape in OV, we summarized the composition of the 22 typical immune cells infiltrating tumor tissues via the CIBERSORT analysis. The results proved that resting Myeloid Dendritic Cells (DCs), memory B cells, and naïve B cells were significantly upregulated in the high-risk group, which was stratified by the anoikis-related signature. Consistent with our findings, Lee and colleagues claimed that DCs played a crucial role in immune responses of OV, in regards to activated memory T cells maintenance, T cell recruitment into tumor tissue, and T cell response initiation [28, 29]. As for B cells, Gupta et al. reported that the tumor microenvironment could induce naïve B cells to differentiate into subsets, such as Bregs, plasma cells, and memory B cells, which played diverse roles in OV progression [30]. A study by Ouyang et al. showed that DCs in the tumor microenvironment could activate B cell differentiation toward the FcγRII low/IL-10 Breg phenotype, which could interrupt the initiation of cancer-induced immunosuppressive events [31]. However, the relationship and underlying mechanism of immune landscapes and anoikis still need further exploration.

Nowadays, regardless of growing advances in immunotherapy and chemotherapy, OV has a high recurrence rate of approximately 70% [4, 32]. Emerging evidence demonstrated that anoikis, a programmed cell death induced by cell detachment from ECM, was a new bridge to tumor immunity, which could influence therapy response [6]. Accordingly, we tried to explore the association between the anoikis pattern and sensitivity to immunotherapy and chemotherapy. Based on the GDSC dataset, high-risk patients were more sensitive to chemotherapy, including Paclitaxel, Vinblastine, Docetaxel, Gemcitabine, and Sorafenib, while less sensitivity to Cisplatin and Bleomycin. Besides, our results revealed that high-risk patients were more likely to benefit from immunotherapies referring to CD274 and PDCD1LG2. Through the TIDE algorithm, we predicted that low-risk OV patients had higher TIDE scores, which represented poorer survival after ICB therapies. We further validated the results in the PRJEB23709 dataset [18], which demonstrated that among patients who received anti-CTLA-4 + anti-PD-1 and anti-PD-1 therapies, the responders had higher Riskscore. The result could hint clinical decision-making, though it still needs to be validated in other immunotherapy datasets.

There were also some limitations in this research. Firstly, the underlying mechanism of the identified ARGs, especially AKT2 and DAPK1, in tumor immune microenvironment and OV progression remained largely unknown, which needs further investigation. Additionally, despite the TCGA and ICGC cohorts, the anoikis-related signature should be validated in more populations, in order to assist clinical decisions for OV precision medicine in the future.

## Conclusion

Briefly, we comprehensively evaluated the importance of the anoikis pattern in OV and filtered ARGs to build a 2-gene prognostic signature (AKT2 and DAPK1) through bioinformatics algorithms. Moreover, we evaluated the tumor immune microenvironment, gene landscape, and sensitivity towards immunotherapy/chemotherapy between risk groups stratified by the anoikis-related signature. Especially, our findings identified the role of DAPK1 in OV metastasis, which presented a potential therapeutic target, though the underlying mechanism needs further investigation.

### Supplementary Information

**Additional file 1: Table S1.** The baseline characteristics of ovarian cancer (OV) patients involved in analysis.

## Data Availability

The data and materials that support the findings are available from corresponding authors upon reasonable requests.

## References

[1] Lheureux S, Braunstein M, Oza AM (2019). Epithelial ovarian cancer: evolution of management in the era of precision medicine. CA Cancer J Clin.

[2] Siegel RL, Miller KD, Wagle NS, Jemal A (2023). Cancer statistics, 2023. CA Cancer J Clin.

[3] Menon U, Karpinskyj C, Gentry-Maharaj A (2018). Ovarian cancer prevention and screening. Obstet Gynecol.

[4] Jacobs IJ, Menon U, Ryan A, Gentry-Maharaj A, Burnell M, Kalsi JK, Amso NN, Apostolidou S, Benjamin E, Cruickshank D (2016). Ovarian cancer screening and mortality in the UK Collaborative Trial of Ovarian Cancer Screening (UKCTOCS): a randomised controlled trial. Lancet.

[5] Raeisi M, Zehtabi M, Velaei K, Fayyazpour P, Aghaei N, Mehdizadeh A (2022). Anoikis in cancer: the role of lipid signaling. Cell Biol Int.

[6] Paoli P, Giannoni E, Chiarugi P (2013). Anoikis molecular pathways and its role in cancer progression. Biochim Biophys Acta.

[7] Sattari Fard F, Jalilzadeh N, Mehdizadeh A, Sajjadian F, Velaei K (2023). Understanding and targeting anoikis in metastasis for cancer therapies. Cell Biol Int.

[8] Adeshakin FO, Adeshakin AO, Afolabi LO, Yan D, Zhang G, Wan X (2021). Mechanisms for modulating anoikis resistance in cancer and the relevance of metabolic reprogramming. Front Oncol.

[9] Taddei ML, Giannoni E, Fiaschi T, Chiarugi P (2012). Anoikis: an emerging hallmark in health and diseases. J Pathol.

[10] Yu Y, Song Y, Cheng L, Chen L, Liu B, Lu D, Li X, Li Y, Lv F, Xing Y (2022). CircCEMIP promotes anoikis-resistance by enhancing protective autophagy in prostate cancer cells. J Exp Clin Cancer Res.

[11] Jin L, Chun J, Pan C, Kumar A, Zhang G, Ha Y, Li D, Alesi GN, Kang Y, Zhou L (2018). The PLAG1-GDH1 axis promotes anoikis resistance and tumor metastasis through CamKK2-AMPK signaling in LKB1-deficient lung cancer. Mol Cell.

[12] Bose M, Sanders A, De C, Zhou R, Lala P, Shwartz S, Mitra B, Brouwer C, Mukherjee P (2023). Targeting tumor-associated MUC1 overcomes anoikis-resistance in pancreatic cancer. Transl Res.

[13] Xu R, Yan Y, Zheng X, Zhang H, Chen W, Li H, Dong Z (2022). Aspirin suppresses breast cancer metastasis to lung by targeting anoikis resistance. Carcinogenesis.

[14] von Mering C, Huynen M, Jaeggi D, Schmidt S, Bork P, Snel B (2003). STRING: a database of predicted functional associations between proteins. Nucleic Acids Res.

[15] Specht E, Kaemmerer D, Sanger J, Wirtz RM, Schulz S, Lupp A (2015). Comparison of immunoreactive score, HER2/neu score and H score for the immunohistochemical evaluation of somatostatin receptors in bronchopulmonary neuroendocrine neoplasms. Histopathology.

[16] Sun T, Ju M, Dai X, Dong H, Gu W, Gao Y, Fu R, Liu X, Huang Y, Liu W (2020). Multilevel defects in the hematopoietic niche in essential thrombocythemia. Haematologica.

[17] Motiño O, Francés DE, Mayoral R, Castro-Sánchez L, Fernández-Velasco M, Boscá L, García-Monzón C, Brea R, Casado M, Agra N (2015). Regulation of microRNA 183 by cyclooxygenase 2 in liver is DEAD-Box helicase p68 (DDX5) dependent: role in insulin signaling. Mol Cell Biol.

[18] Qian J, Olbrecht S, Boeckx B, Vos H, Laoui D, Etlioglu E, Wauters E, Pomella V, Verbandt S, Busschaert P (2020). A pan-cancer blueprint of the heterogeneous tumor microenvironment revealed by single-cell profiling. Cell Res.

[19] Robert L, Ribas A, Hu-Lieskovan S (2016). Combining targeted therapy with immunotherapy. Can 1+1 equal more than 2?. Semin Immunol.

[20] Gide TN, Quek C, Menzies AM, Tasker AT, Shang P, Holst J, Madore J, Lim SY, Velickovic R, Wongchenko M (2019). Distinct immune cell populations define response to anti-PD-1 monotherapy and anti-PD-1/anti-CTLA-4 combined therapy. Cancer Cell.

[21] Qian S, Wen Y, Mei L, Zhu X, Zhang H, Xu C (2023). Development and validation of a novel anoikis-related gene signature for predicting prognosis in ovarian cancer. Aging (Albany NY).

[22] Cariaga-Martinez AE, Lopez-Ruiz P, Nombela-Blanco MP, Motino O, Gonzalez-Corpas A, Rodriguez-Ubreva J, Lobo MV, Cortes MA, Colas B (2013). Distinct and specific roles of AKT1 and AKT2 in androgen-sensitive and androgen-independent prostate cancer cells. Cell Signal.

[23] Moro L, Arbini AA, Yao JL, di Sant'Agnese PA, Marra E, Greco M (2009). Mitochondrial DNA depletion in prostate epithelial cells promotes anoikis resistance and invasion through activation of PI3K/Akt2. Cell Death Differ.

[24] Fujio Y, Mitsuuchi Y, Testa JR, Walsh K (2001). Activation of Akt2 Inhibits anoikis and apoptosis induced by myogenic differentiation. Cell Death Differ.

[25] Zheng B, Geng L, Zeng L, Liu F, Huang Q (2018). AKT2 contributes to increase ovarian cancer cell migration and invasion through the AKT2-PKM2-STAT3/NF-κB axis. Cell Signal.

[26] Qin R, Melamed S, Yang B, Saxena M, Sheetz MP, Wolfenson H (2022). Tumor suppressor DAPK1 catalyzes adhesion assembly on rigid but anoikis on soft matrices. Front Cell Dev Biol.

[27] Kwon T, Youn H, Son B, Kim D, Seong KM, Park S, Kim W, Youn B (2016). DANGER is involved in high glucose-induced radioresistance through inhibiting DAPK-mediated anoikis in non-small cell lung cancer. Oncotarget.

[28] Lee YS, Radford KJ (2019). The role of dendritic cells in cancer. Int Rev Cell Mol Biol.

[29] Sabado RL, Balan S, Bhardwaj N (2017). Dendritic cell-based immunotherapy. Cell Res.

[30] Gupta P, Chen C, Chaluvally-Raghavan P, Pradeep S (2019). B cells as an immune-regulatory signature in ovarian cancer. Cancers (Basel).

[31] Ouyang FZ, Wu RQ, Wei Y, Liu RX, Yang D, Xiao X, Zheng L, Li B, Lao XM, Kuang DM (2016). Dendritic cell-elicited B-cell activation fosters immune privilege via IL-10 signals in hepatocellular carcinoma. Nat Commun.

[32] Webb PM, Jordan SJ (2017). Epidemiology of epithelial ovarian cancer. Best Pract Res Clin Obstet Gynaecol.

